# The Law Regulating the Practice of Dentistry in the State of Virginia

**Published:** 1886-04

**Authors:** 


ARTICLE V.

### THE LAW REGULATING THE PRACTICE OF DENTISTRY IN THE STATE OF VIRGINIA.

Approved February 26th, 1886.

1. Be it enacted by the general assembly of Virginia, That from and after the passage of this act it shall be unlawful for any person, except regularly authorized physicians and surgeons, to engage in the practice of dentistry in the commonwealth of Virginia, or to receive license from any commissioner of the revenue, unless such person has graduated and received a diploma from the faculty of a reputable institution where this specialty is taught, and chartered under the authority of some one of the United States, or of a foreign government, acknowledged as such, or shall have obtained a certificate from a board of examiners duly appointed, and authorized by the provisions of this act to issue such certificates; provided, that nothing herein contained shall prevent any person from extracting teeth for any one suffering from toothache.

2. That the board of examiners shall consist of six practitioners of dentistry, who are of acknowledged ability in the profession. Said board shall be appointed by the governor, who shall select from twelve candidates named by the Virginia State Dental Association at their next annual meeting, of whom two shall serve one year, two for two years, and two for three years, and to reside in different sections of the state; and each year thereafter two shall be appointed in the same manner from four nominees, to serve for three years, or until their successors are elected. All vacancies for unexpired terms shall be filled by the governor from names furnished him by the board.

3. That it shall be the duty of this board:

First. To meet annually at the time and place of meeting of the Virginia State Dental Association, and at such other time and place as the said board shall agree upon, to conduct the examination of applicants. They shall also meet for the same purpose at the call of any four members of said board, at such time and place as may be designated. Thirty days' notice must be given of the meetings, by advertising in at least two of the daily papers published in the commonwealth of Virginia.

Second. To grant a certificate of ability to practice dentistry, which certificate shall be signed by said board, and stamped with a suitable seal, to all applicants who undergo a satisfactory examination, and who received at least four affirmative votes.

Third. To keep a book in which shall be registered the names and qualifications of each, as far as practicable, of all persons who have been granted certificates of ability, to practice dentistry under the provisions of this act.

4. That the book so kept shall be a book of record, and transcripts from it certified to by the officer who has it in keeping, with the seal of said board of examiners, shall be evidence in any court of this commonwealth.

5. That four members of this board shall constitute a quorum for the transaction of business; and should a quorum not be present on any day appointed for their meeting, those present may adjourn from time to time until a quorum is present.

6. That any person who shall, in violation of this act, practice dentistry in the commonwealth of Virginia, shall be liable to indictment in the circuit, county or corporation courts; and on conviction, shall be fined not less than fifty nor more than two hundred dollars: provided, that any person so convicted shall not be entitled to any fee for services rendered; and if a fee shall have been paid the patient, or his or her heirs, may recover the same as debts of like amount are now recovered by law.

7. That all fines collected shall inure to the public school fund of the county or corporation in which the prosecution occurs.

8. That nothing in this act shall apply to persons who shall be engaged in the practice of dentistry in this commonwealth at the time of or prior to the passage of this act.

9. To provide a fund to carry out the provisions of the third section of this act, it shall be the duty of said board of examiners to collect from those who shall appear before them for examination the sum of ten dollars each.

10. This act shall be in force from its passage.

